# CTNNA3 genetic polymorphism may be a new genetic signal of type 2 diabetes in the Chinese Han population: a case control study

**DOI:** 10.1186/s12920-021-01105-8

**Published:** 2021-10-30

**Authors:** Yunjun Zhang, Xiaoman Zhou, Wanjuan Dai, Juan Sun, Mei Lin, Yutian Zhang, Yipeng Ding

**Affiliations:** 1 Department of General Practice, Hainan General Hospital, #19, Xiuhua Road, Xiuying District, Haikou, 570311 Hainan People’s Republic of China; 2 Health Center, Hainan General Hospital, Haikou, 570311 Hainan People’s Republic of China

**Keywords:** Type 2 diabetes, Case–control study, *CTNNA3*, Single nucleotide polymorphisms

## Abstract

**Background:**

Type 2 Diabetes (T2D) is the result of a combination of genes and environment. The identified genetic loci can only explain part of T2D risk. Our study is aimed to explore the association between CTNNA3 single nucleotide polymorphisms (SNPs) and T2D risk.

**Methods:**

We conducted a 'case–control' study among 1002 Chinese Han participants. Four candidate SNPs of CTNNA3 were selected (rs10822745 C/T, rs7920624 A/T, rs2441727 A/G, rs7914287 A/G), and logistic regression analysis was used to evaluate the association between candidate SNPs and T2D risk. We used single factor analysis of variance to analyze the differences of clinical characteristics among different genotypes. In this study, haplotype analysis was conducted by plink1.07 and Haploview software and linkage disequilibrium (LD) was calculated. The interaction of candidate SNPs in T2D risk was evaluated by multi-factor dimensionality reduction (MDR). Finally, we conducted a false-positive report probability (FPRP) analysis to detect whether the significant findings were just chance or noteworthy observations.

**Results:**

The results showed that *CTNNA3*-rs7914287 was a risk factor for T2D (‘T’: OR = 1.33, *p* = 0.003; ‘TT’: OR = 2.21, *p* = 0.001; ‘TT’ (recessive): OR = 2.09, *p* = 0.001; Log-additive: OR = 1.34, *p* = 0.003). The results of subgroup analysis showed that rs7914287 was significantly associated with the increased risk of T2D among participants who were older than 60 years, males, smoking, drinking, or BMI > 24. We also found that rs2441727 was associated with reducing the T2D risk among participants who were older than 60 years, smoking, or drinking. In addition, rs7914287 was associated with T2D patients with no retinal degeneration; rs10822745 and rs7920624 were associated with the course of T2D patients. High density lipoprotein levels had significant differences under different genotypes of rs10822745. Under the different genotypes of rs7914287, the levels of aspartate aminotransferase, alanine aminotransferase and gamma-glutamyltransferase were also significantly different.

**Conclusion:**

We found that CTNNA3 genetic polymorphisms can be used as a new genetic signal of T2D risk in Chinese Han population. Especially, *CTNNA3*-rs7914287 showed an outstanding and significant association with T2D risk in both overall analysis and subgroup analysis.

**Supplementary Information:**

The online version contains supplementary material available at 10.1186/s12920-021-01105-8.

## Introduction

Type 2 diabetes (T2D) is non-insulin-dependent diabetes, is a form of diabetes is much more prevalent.The prevalence of T2D is getting higher and higher worldwide. T2D and its complications have reached the level of an epidemic, which has attracted great attention in the field of scientific research [1]. A number of studies have confirmed that T2D is a complex disease affected by multiple factors and is the result of the interaction between genes and the environment [1–4]. Evidence from genetic epidemiology indicates that the occurrence of type 2 diabetes has a strong genetic basis [5–8]. In recent years, with the improvement and application of molecular epidemiology or genetic testing technology, some T2D-related genetic loci have been identified one after another [9–15], but it can only explain the T2D risk among part of population [1, 16]. Therefore, it is necessary to discover new genetic signals of T2D in different populations, which will provide valuable references for clinical diagnosis and early prevention of T2D.

*CTNNA3* can encode αT-catenin protein and is a member of the α-catenin family of cell–cell adhesion molecules [17]. Chiarella, S. E., et al. have proposed that *CTNNA3* may be the most relevant type of α-catenin in human diseases [18]. In a recent genome-wide association study on Metabolic Syndrome of African Lineage (MetS), single nucleotide polymorphism (SNPs) of *CTNNA3* were reported can be new genetic signals of metabolic syndrome risk. There have been studies have reported that people with MetS have at least a five-fold increase in the risk of T2D [19, 20]. But so far, there is no report about the association between *CTNNA3* SNPs and T2D risk.

Therefore, in order to explore the association between *CTNNA3* SNPs and T2D risk, we conducted a 'case–control' study among a total of 1,002 Chinese Han population. We not only conducted an overall analysis, but also divided participants according to the known potential environmental risk factors of T2D for subgroup analysis, such as drinking [21], smoking [22], age [23], etc. Finally, the association between candidate SNPs and T2D risk will be evaluated. Our study will provide data supplement for genetic loci associated with T2D risk in Chinese Han population, and lay a certain theoretical foundation for the individualized prevention and treatment of T2D.

## Materials and methods

### Study subjects

In this study, a ‘case–control’ study design was used to analyze the association between SNPs and T2D risk among 1002 participants (501 cases and 501 controls). *Case group*: (1) T2D patients who are outpatients or hospitalized in Hainan General Hospital. (2) Patients who were diagnosed as T2D for the first time or who have been clearly diagnosed as T2D (Fasting blood glucose (FBG) ≥ 7.0 mmol/L, OGTT 2 h blood glucose ≥ 11.1 mmol/L or random blood glucose ≥ 11.1 mmol/L). (3) No history of other complicated diseases (malignant tumors, cardiovascular disease history, etc.). (4) No history of genetic diseases. *Control group*: (1) Healthy individuals undergoing physical examination at the same hospital's health examination center during the same period. (2) FBG ≤ 6.1 mmol/L. (3) No complicated chronic diseases, and tumor patients or people with tumor history are excluded. (4) Recruit healthy individuals who match the case group in terms of age and gender (excluding confounding factors caused by differences in the distribution of exposure factors in the case/control group).

In this study, questionnaire survey on demographic and epidemiological information among all participants was conducted by a professional doctor. The contents of the questionnaire include gender, height, weight, smoking/drinking status, whether diabetic patients were accompanied by retinopathy, and the course of T2D. After obtaining the informed consent of all participants, we collected their peripheral blood samples for subsequent DNA extraction. The study was approved by the ethics committee of Hainan General Hospital.

### Selection of SNPs

The specific steps for selecting SNPs are as follows: (1) We obtained the physical position of the *CTNNA3* on the Chromosome 10: 65,912,457–67,763,637 through the e!GRCh37 database (http://asia.ensembl.org/Homo_sapiens/Info/Index). In the VCF to PED Converter window (http://grch37.ensembl.org/Homo_sapiens/Tools/VcftoPed), we entered the gene location, selected the CHB and CHS population, and downloaded the ped and info file for the variations of *CTNNA3*. (2) Then we used Haploview software for quality control (HWE > 0.01, MAF > 0.05, Min Genotype > 75%, and Tagger r^2^ > 0.8) to select tagSNP. Finally, four SNPs of *CTNNA3* (rs10822745 C/T, rs7920624 A/T, rs2441727 A/G, rs7914287 A/G) were selected for our study.’ You can check the revisions from the ‘Methods-Selection of SNPs.

### DNA extraction and genotyping

DNA extraction and purification were performed according to the instructions of the kit (GoldMag Co. Ltd. Xi’an, China). We store the purified DNA in an ultra-low temperature refrigerator (−80 ℃) until needed in the next experiment. We used MassARRAY Assay Design software to design all primers we need. The genotyping in this study was conducted by the MassARRAY system (Agena, San Diego, CA, USA).

In order to reduce experimental errors and ensure the reliability and repeatability of experimental results, we randomly select 10% of DNA samples for repeatability testing. The repetition rate of experimental results needs to be > 99%.

### Statistical analysis

The differences in demographic characteristics (age, gender, BMI, etc.) were tested by SPSS version 21.0 software (SPSS, Chicago, IL, USA) (χ^2^ test/*t*-test). After testing whether the four candidate genetic loci of CTNNA3 meet Hardy–Weinberg equilibrium (SPSS version 21.0 software), we used logistic regression model to calculate the odds ratio (OR) and 95% confidence interval (CI). Then, according to the value of OR and CI, the association between CTNNA3 candidate SNPs and T2D risk was estimated (OR value represents relative risk; OR = 1: this factor has no effect on T2D risk; OR < 1: T2D protective factor; OR > 1: T2D risk factor). Using the wild-type allele as a reference, the online tool software plink 1.07 was used to estimate multiple genetic models. The statistical results obtained were adjusted by age and gender, and all tests were two-sided tests. In addition, we conducted a false-positive report probability (FPRP) analysis to detect whether the significant findings were just chance or noteworthy observations [24]. In this study, haplotype analysis was conducted by plink1.07 and Haploview software and linkage disequilibrium (LD) was calculated. Finally, the interaction of candidate SNPs in T2D risk was evaluated by multi-factor dimensionality reduction (MDR).

## Results

### Sample overview and collection

There was no genetic relationship among all participants in our study. Among them, the average age of T2D patients was 59.86 ± 12.86 years old, males 359 (72%), females 142 (28%). The average age of healthy individuals was 59.60 ± 10.09 years, males 359 (72%), females 142 (28%). The basic demographic and epidemiological information was shown in Table 1. There was no statistical difference between the case and the control group in gender (*p* = 0.528) or age (*p* = 0.714).

**Table 1:** Characteristics of patients with type 2 diabetes and healthy individuals

Characteristics		Cases	Control	*P*
		n = 501	n = 501	
Age (years)	Mean ± SD	59.86 ± 12.86	59.60 ± 10.09	0.714
	** > **60	240 (48%)	262 (52%)	
	≤ 60	261 (52%)	239 (48%)	
Gender	Male	359 (72%)	359 (72%)	0.528
	Female	142 (28%)	142 (28%)	
Course of disease	> 10 versus ≤ 10 years	194 (39%)	306 (61%)	
No retinal degeneration	Yes	70 (14%)	–	
	No	240 (48%)	–	
Smoking	Yes	218 (44%)	124 (25%)	0.593
	No	281 (56%)	173 (35%)	
Drinking	Yes	109 (22%)	127 (25%)	< 0.0001
	No	385 (77%)	143 (29%)	
BMI (kg/m^2^)	BMI > 24	239 (48%)	187 (37%)	0.089
	BMI ≤ 24	203 (41%)	123 (25%)	

Course of disease: the length of time the case has suffered from T2D (participants are divided by the average of the length of time)

BMI, Body mass index

### Genotyping and information about candidate SNPs

The 4 candidate genetic loci of *CNNTA3* (rs10822745 C/T, rs7920624 A/T, rs2441727 A/G, rs7914287 A/G) were successfully genotyped. As shown in Table 2, all candidate SNPs met HWE (*p* > 5%). The results of HaploReg showed that the candidate SNPs were regulated by a variety of factors, and the specific factors were detailed in Table 2.

**Table 2:** The basic information and HWE about the selected SNPs of *CTNNA3*

Gene	SNP ID	Function	Chr: position	Alleles (A/B)	MAF		HWE (*P* value)	Haploreg 4.1
					Cases	Controls		
CTNNA3	rs10822745	Intronic	10: 66,194,307	C/T	0.429	0.417	0.646	Motifs changed
CTNNA3	rs7920624	Intronic	10: 66,203,428	A/T	0.488	0.498	0.929	Motifs changed
CTNNA3	rs2441727	Intronic	10: 66,465,128	A/G	0.182	0.204	0.406	NHGRI/EBI GWAS hits
CTNNA3	rs7914287	Intronic	10: 67,590,805	T/C	0.355	0.292	0.124	–

HWE, Hardy–Weinberg equilibrium; Alleles (A/B), minor/major allele; SNP, single nucleotide polymorphisms; MAF, minor allele frequency

*p* > 0.05 indicates that the genotypes were in Hardy–Weinberg Equilibrium

### Evaluation of association between candidate SNPs and T2D risk (overall analysis)

The evaluation results of the association between candidate SNPs and T2D risk (Table 3) showed that only *CNNTA3* rs7914287 had a significant association with the T2D risk among participants. Specifically, rs7914287 can significantly increase the T2D risk under allele (T vs. C: OR = 1.33, CI 1.10–1.61, *p* = 0.003), homozygous (TT vs. CC: OR = 2.21, CI 1.41–3.47, *p* = 0.001), recessive (TT vs. TC-CC: OR = 2.09, CI 1.36–3.22, *p* = 0.001), and log-additive models (OR = 1.34, CI 1.10–1.62, *p* = 0.003). We did not find any evidence that the remaining three candidate SNPs were associated with the T2D risk.

**Table 3:** Analysis of the association between susceptibility of type 2 diabetes and single nucleotide polymorphism of *CTNNA3*

SNP ID	Model	Genotype	Case	Control	Adjusted by age and gender	
					OR (95% CI)	*p*
rs10822745	Allele	C	430 (42.91%)	417 (41.70%)	1.05 (0.88–1.26)	0.582
		T	572 (57.09%)	583 (58.30%)	1.00	
	Genotype	CC	87 (17.4%)	84 (16.8%)	1.10 (0.76–1.59)	0.631
		CT	256 (51.1%)	249 (49.8%)	1.09 (0.82–1.44)	0.558
		TT	158 (31.5%)	167 (33.4%)	1.00	
	Dominant	CC-CT	343 (68.5%)	333 (66.6%)	1.09 (0.84–1.42)	0.528
		TT	158 (31.5%)	167 (33.4%)	1.00	
	Recessive	CC	87 (17.4%)	84 (16.8%)	1.04 (0.75–1.45)	0.811
		CT-TT	414 (82.6%)	416 (83.2%)	1.00	
	Log-additive	–	–	–	1.05 (0.88–1.26)	0.575
rs7920624	Allele	A	485 (48.79%)	498 (49.80%)	0.96 (0.81–1.15)	0.653
		T	509 (51.21%)	502 (50.20%)	1.00	
	Genotype	AA	110 (22.1%)	123 (24.6%)	0.91 (0.64–1.31)	0.623
		AT	265 (53.3%)	252 (50.4%)	1.08 (0.80–1.46)	0.629
		TT	122 (24.6%)	125 (25%)	1.00	
	Dominant	AA-AT	375 (75.5%)	375 (75%)	1.02 (0.77–1.37)	0.872
		TT	122 (24.6%)	125 (25%)	1.00	
	Recessive	AA	110 (22.1%)	123 (24.6%)	0.87 (0.65–1.17)	0.349
		AT-TT	387 (77.9%)	377 (75.4%)	1.00	
	Log-additive	–	–	–	0.96 (0.80–1.15)	0.637
rs2441727	Allele	A	182 (18.16%)	202 (20.45%)	0.86 (0.69–1.08)	0.197
		G	820 (81.84%)	786 (79.55%)	1.00	
	Genotype	AA	17 (3.4%)	17 (3.4%)	0.91 (0.46–1.83)	0.802
		AG	148 (29.5%)	168 (34%)	0.81 (0.62–1.06)	0.128
		GG	336 (67.1%)	309 (62.5%)	1.00	
	Dominant	AA-AG	165 (32.9%)	185 (37.5%)	0.82 (0.63–1.07)	0.137
		GG	336 (67.1%)	309 (62.5%)	1.00	
	Recessive	AA	17 (3.4%)	17 (3.4%)	0.98 (0.49–1.95)	0.955
		AG-GG	484 (96.6%)	477 (96.6%)	1.00	
	Log-additive	–	–	–	0.86 (0.69–1.08)	0.192
rs7914287	Allele	T	355 (35.50%)	284 (29.22%)	1.33 (1.10–1.61)	0.003*
		C	645 (64.50%)	688 (70.78%)	1.00	
	Genotype	TT	68 (13.6%)	34 (7%)	2.21 (1.41–3.47)	0.001*
		TC	219 (43.8%)	216 (44.4%)	1.12 (0.86–1.46)	0.389
		CC	213 (42.6%)	236 (48.6%)	1.00	
	Dominant	TT-TC	287 (57.4%)	250 (51.4%)	1.27 (0.99–1.63)	0.062
		CC	213 (42.6%)	236 (48.6%)	1.00	
	Recessive	TT	68 (13.6%)	34 (7%)	2.09 (1.36–3.22)	0.001*
		TC-CC	432 (86.4%)	452 (93%)	1.00	
	Log-additive	–	–	–	1.34 (1.10–1.62)	0.003*

SNP, Single nucleotide polymorphisms; OR, odds ratio; CI, Confidence interval

*p* < 0.05, bold text and '*' indicate statistical significance

“–” indicates Log-additive model

### Evaluation of association between candidate SNPs and T2D risk (subgroup analysis)

*Age and gender* The results showed (Table 4) that *CNNTA3* rs2441727 can significantly reduce the T2D risk in participants who were aged ≤ 60 years old under multiple genetic models (heterozygote: OR = 0.58, CI 0.39–0.87,* p* = 0.008; dominant: OR = 0.64, CI 0.44–0.93, *p* = 0.021). rs7914287 was not only a risk factor for T2D in participants aged > 60 years old (allele: OR = 1.37, CI 1.06–1.79, *p* = 0.018; homozygote: OR = 2.22, CI 1.18–4.17, *p* = 0.013; dominant: OR = 1.56, CI 1.08–2.25, *p* = 0.019; recessive: OR = 1.85, CI 1.02–3.35, *p* = 0.044; log-additive: OR = 1.47, CI 1.11–1.94, *p* = 0.007), but also a risk factor for T2D in participants aged ≤ 60 years old (homozygote: OR = 2.71, CI 1.36–5.42, *p* = 0.005; recessive: OR = 2.73, CI 1.40–5.32, *p* = 0.003; log-additive: OR = 1.33, CI 1.01–1.75, *p* = 0.041). In the gender stratification analysis, rs7914287 can significantly increase the T2D risk in male participants (Allele: OR = 2.33, CI 1.07–3.67, *p* = 0.012; homozygote: OR = 2.20, CI 1.29–3.77, *p* = 0.004; recessive: OR = 2.08, CI 1.24–3.49, *p* = 0.005; log-additive: OR = 2.33, CI 1.06–3.67, *p* = 0.013).

**Table 4:** The SNPs of *CTNNA3* associated with susceptibility of type 2 diabetes in the subgroup tests (age and gender)

SNP ID	Model	Genotype	Age, years				Gender			
			OR (95% CI)	*p*	OR (95% CI)	*P*	OR (95% CI)	*p*	OR (95% CI)	*p*
			≤ 60 (N = 500)		> 60 (N = 502)		Female (N = 284)		Male (N = 718)	
rs10822745	Allele	C	1.03 (0.80–1.32)	0.844	1.07 (0.83–1.38)	0.587	1.09 (0.78–1.53)	0.600	1.04 (0.84–1.28)	0.749
		T	1.00		1.00		1.00		1.00	
	Genotype	CC	1.09 (0.65–1.85)	0.739	1.05 (0.61–1.79)	0.872	1.20 (0.61–2.36)	0.601	1.05 (0.68–1.64)	0.816
		CT	0.91 (0.61–1.36)	0.660	1.21 (0.81–1.81)	0.348	1.08 (0.64–1.82)	0.775	1.09 (0.78–1.52)	0.613
		TT	1.00		1.00		1.00		1.00	
	Dominant	CC-CT	0.96 (0.65–1.40)	0.819	1.17 (0.8–1.71)	0.426	1.11 (0.68–1.82)	0.675	1.08 (0.79–1.48)	0.630
		TT	1.00		1.00		1.00		1.00	
	Recessive	CC	1.16 (0.73–1.84)	0.541	0.93 (0.58–1.51)	0.771	1.15 (0.62–2.11)	0.659	1.00 (0.68–1.48)	0.999
		CT-TT	1.00		1.00		1.00		1.00	
	Log-additive	–	1.02 (0.79–1.32)	0.854	1.05 (0.81–1.37)	0.699	1.09 (0.78–1.52)	0.601	1.04 (0.84–1.29)	0.742
rs7920624	Allele	A	1.13 (0.88–1.45)	0.329	1.05 (0.82–1.35)	0.700	0.95 (0.68–1.31)	0.737	0.97 (0.79–1.19)	0.749
		T	1.00		1.00		1.00		1.00	
	Genotype	AA	1.28 (0.76–2.14)	0.350	1.09 (0.65–1.83)	0.745	0.89 (0.46–1.73)	0.727	0.93 (0.60–1.42)	0.720
		AT	1.02 (0.65–1.59)	0.949	1.47 (0.94–2.29)	0.092	1.00 (0.57–1.76)	1.000	1.11 (0.78–1.59)	0.567
		TT	1.00		1.00		1.00		1.00	
	Dominant	AA-AT	1.09 (0.72–1.67)	0.679	1.33 (0.87–2.03)	0.183	0.96 (0.57–1.64)	0.890	1.05 (0.75–1.48)	0.779
		TT	1.00		1.00		1.00		1.00	
	Recessive	AA	1.27 (0.84–1.90)	0.259	0.84 (0.55–1.28)	0.411	0.89 (0.51–1.54)	0.673	0.86 (0.61–1.22)	0.399
		AT-TT	1.00		1.00		1.00		1.00	
	Log-additive	–	1.14 (0.88–1.47)	0.336	1.04 (0.81–1.35)	0.755	0.94 (0.68–1.32)	0.733	0.96 (0.78–1.19)	0.733
rs2441727	Allele	A	0.98 (0.71–1.35)	0.887	0.78 (0.58–1.07)	0.122	0.89 (0.60–1.33)	0.581	0.85 (0.65–1.11)	0.238
		G	1.00		1.00		1.00		1.00	
	Genotype	AA	0.46 (0.11–1.88)	0.281	0.73 (0.49–1.67)	0.748	0.95 (0.36–2.03)	0.933	0.82 (0.33–2.06)	0.677
		AG	0.86 (0.43–1.26)	0.757	0.58 (0.39–0.87)	0.008*	0.80 (0.48–1.31)	0.371	0.82 (0.59–1.13)	0.218
		GG	1.00		1.00		1.00		1.00	
	Dominant	AA-AG	0.84 (0.70–1.48)	0.931	0.64 (0.44–0.93)	0.021*	0.83 (0.51–1.33)	0.433	0.82 (0.60–1.12)	0.206
		GG	1.00		1.00		1.00		1.00	
	Recessive	AA	0.45 (0.11–1.84)	0.267	0.57 (0.60–0.76)	0.455	0.94 (0.40–1.23)	0.812	0.88 (0.35–2.19)	0.775
		AG-GG	1.00		1.00		1.00		1.00	
	Log-additive	–	0.96 (0.68–1.36)	0.830	0.77 (0.57–1.05)	0.103	0.89 (0.60–1.33)	0.579	0.84 (0.64–1.11)	0.228
rs7914287	Allele	T	1.31 (1.00–1.72)	0.052	1.37 (1.06–1.79)	0.018*	1.34 (0.94–1.90)	0.107	2.33 (1.07–3.67)	0.012*
		C	1.00		1.00		1.00		1.00	
	Genotype	TT	2.71 (1.36–5.42)	0.057	2.22 (1.18–4.17)	0.013*	2.26 (0.97–5.22)	0.058	2.20 (1.29–3.77)	0.004*
		TC	0.99 (0.68–1.44)	0.952	1.43 (0.97–2.11)	0.070	1.11 (0.67–1.83)	0.681	1.12 (0.82–1.54)	0.461
		CC	1.00		1.00		1.00		1.00	
	Dominant	TT-TC	1.18 (0.82–1.68)	0.372	1.56 (1.08–2.25)	0.019*	1.27 (0.79–2.04)	0.330	1.27 (0.95–1.71)	0.113
		CC	1.00		1.00		1.00		1.00	
	Recessive	TT	2.73 (1.40–5.32)	0.120	1.85 (1.02–3.35)	0.044*	2.14 (0.96–4.76)	0.063	2.08 (1.24–3.49)	0.005*
		TC-CC	1.00		1.00		1.00		1.00	
	Log-additive	–	1.33 (1.01–1.75)	0.064	1.47 (1.11–1.94)	0.007*	1.35 (0.94–1.93)	0.106	2.33 (1.06–3.67)	0.013*

SNP, Single nucleotide polymorphisms; OR, odds ratio; CI, Confidence interval

*p* < 0.05, bold text and '*' indicate statistical significance

“–” indicates Log-additive model

*Smoking and drinking* The results showed (Table 5) that rs2441727 significantly reduced the T2D risk in participants with a history of smoking (heterozygote: OR = 0.61, CI 0.41–0.92, *p* = 0.018; dominant: OR = 0.65, CI 0.44–0.97, *p* = 0.034). At the same time, rs2441727 reduced the T2D risk in drinking participants under the heterozygous genetic model (heterozygote: OR = 0.63, CI 0.42–0.94,* p* = 0.025). rs7914287 was a risk factor for T2D in smoking (allele: OR = 2.36, CI 1.01–4.82, *p* = 0.023; homozygote: OR = 2.50, CI 1.18–5.32, *p* = 0.017; recessive: OR = 2.44, CI 1.18–5.05, *p* = 0.016) and drinking participants (allele: OR = 1.41, CI 1.04–2.91, *p* = 0.025; homozygote: OR = 2.11, CI 1.34–5.20, *p* = 0.008; recessive: OR = 2.00, CI 1.33–4.77, *p* = 0.008).

**Table 5:** The SNPs of *CTNNA3* associated with susceptibility of type 2 diabetes in the subgroup tests (smoking and drinking)

SNP ID	Model	Genotype	Smoking				Drinking			
			OR (95% CI)	*p*	OR (95% CI)	*p*	OR (95% CI)	*p*	OR (95% CI)	*p*
			No (N = 454)		Yes (N = 342)		No (N = 528)		Yes (N = 236)	
rs10822745	Allele	C	0.96 (0.70–1.31)	0.777	1.11 (0.85–1.46)	0.447	1.13 (0.78–1.63)	0.524	1.05 (0.80–1.39)	0.715
		T	1.00		1.00		1.00		1.00	
	Genotype	CC	0.79 (0.40–1.56)	0.500	1.26 (0.73–2.17)	0.407	1.26 (0.57–2.76)	0.568	0.98 (0.57–1.69)	0.954
		CT	1.25 (0.76–2.05)	0.377	1.11 (0.72–1.71)	0.628	1.21 (0.68–2.14)	0.518	1.43 (0.93–2.21)	0.107
		TT	1.00		1.00		1.00		1.00	
	Dominant	CC-CT	1.13 (0.70–1.8)	0.619	1.15 (0.77–1.73)	0.488	1.22 (0.71–2.11)	0.476	1.28 (0.86–1.91)	0.230
		TT	1.00		1.00		1.00		1.00	
	Recessive	CC	0.69 (0.38–1.27)	0.233	1.18 (0.73–1.91)	0.496	1.12 (0.55–2.27)	0.750	0.80 (0.50–1.30)	0.375
		CT-TT	1.00		1.00		1.00		1.00	
	Log-additive	–	0.95 (0.68–1.33)	0.773	1.12 (0.86–1.46)	0.403	1.14 (0.78–1.66)	0.505	1.05 (0.79–1.37)	0.753
rs7920624	Allele	A	1.01 (0.74–1.39)	0.931	0.95 (0.73–1.25)	0.733	1.10 (0.77–1.59)	0.598	0.99 (0.75–1.29)	0.921
		T	1.00		1.00		1.00		1.00	
	Genotype	AA	1.03 (0.54–1.97)	0.918	0.89 (0.52–1.51)	0.659	1.23 (0.60–2.53)	0.574	0.97 (0.56–1.65)	0.899
		AT	1.52 (0.88–2.63)	0.131	1.02 (0.65–1.60)	0.943	1.36 (0.73–2.55)	0.334	1.37 (0.86–2.19)	0.182
		TT	1.00		1.00		1.00		1.00	
	Dominant	AA-AT	1.36 (0.81–2.27)	0.249	0.97 (0.63–1.49)	0.898	1.31 (0.73–2.36)	0.361	1.22 (0.79–1.89)	0.363
		TT	1.00		1.00		1.00		1.00	
	Recessive	AA	0.77 (0.46–1.31)	0.342	0.88 (0.56–1.37)	0.567	1.01 (0.56–1.83)	0.978	0.78 (0.50–1.23)	0.286
		AT-TT	1.00		1.00		1.00		1.00	
	Log-additive	–	1.02 (0.74–1.42)	0.892	0.94 (0.72–1.23)	0.674	1.11 (0.78–1.59)	0.563	0.99 (0.75–1.31)	0.937
rs2441727	Allele	A	1.01 (0.67–1.53)	0.953	0.79 (0.56–1.10)	0.154	0.97 (0.62–1.51)	0.896	0.80 (0.57–1.12)	0.191
		G	1.00		1.00		1.00		1.00	
	Genotype	AA	3.08 (0.36–6.41)	0.305	1.32 (0.41–4.29)	0.645	0.67 (0.15–2.95)	0.599	2.60 (0.57–5.82)	0.215
		AG	0.88 (0.55–1.42)	0.607	0.61 (0.41–0.92)	0.018*	1.08 (0.63–1.87)	0.778	0.63 (0.42–0.94)	0.025*
		GG	1.00		1.00		1.00		1.00	
	Dominant	AA-AG	0.94 (0.58–1.50)	0.780	0.65 (0.44–0.97)	0.034*	1.04 (0.61–1.76)	0.892	0.69 (0.46–1.03)	0.071
		GG	1.00		1.00		1.00		1.00	
	Recessive	AA	0.82 (0.38–1.34)	0.287	0.57 (0.49–1.06)	0.448	0.65 (0.15–2.83)	0.570	0.27 (0.68–1.83)	0.144
		AG-GG	1.00		1.00		1.00		1.00	
	Log-additive	–	0.93 (0.66–1.55)	0.963	0.75 (0.54–1.06)	0.108	0.98 (0.62–1.56)	0.947	0.83 (0.58–1.17)	0.281
rs7914287	Allele	T	1.36 (0.97–1.91)	0.078	2.36 (1.01–4.82)	0.023*	1.20 (0.81–1.77)	0.368	1.41 (1.04–2.91)	0.025*
		C	1.00		1.00		1.00		1.00	
	Genotype	TT	1.91 (0.87–4.21)	0.109	2.50 (1.18–5.32)	0.017*	1.33 (0.55–3.20)	0.527	2.11 (1.34–5.20)	0.008*
		TC	1.29 (0.80–2.07)	0.302	1.06 (0.71–1.58)	0.788	1.21 (0.70–2.09)	0.501	1.08 (0.72–1.62)	0.721
		CC	1.00		1.00		1.00		1.00	
	Dominant	TT-TC	1.39 (0.89–2.19)	0.152	1.23 (0.83–1.81)	0.299	1.23 (0.73–2.07)	0.436	1.28 (0.86–1.90)	0.219
		CC	1.00		1.00		1.00		1.00	
	Recessive	TT	1.69 (0.79–3.60)	0.175	2.44 (1.18–5.05)	0.016*	1.21 (0.52–2.79)	0.655	2.00 (1.33–4.77)	0.008*
		TC-CC	1.00		1.00		1.00		1.00	
	Log-additive	–	1.35 (0.96–1.89)	0.089	1.34 (0.99–1.80)	0.055	1.17 (0.79–1.73)	0.428	1.41 (1.04–1.91)	0.027*

OR, odds ratio; CI, confidence interval

*p* < 0.05, bold text and '*' indicate statistical significance

“–” indicates Log-additive model

*BMI* The results showed (Table 6) that rs7914287 was a risk factor for T2D no matter in the participants with BMI ≤ 24 or BMI > 24. Specifically, among the participants with BMI ≤ 24, rs7914287 can significantly increase the T2D risk under allele (OR = 1.57, CI 1.11–2.24, *p* = 0.011), homozygote (OR = 3.66, CI 1.43–5.36, *p* = 0.007), recessive (OR = 2.25, CI 1.31–4.07, *p* = 0.011), and log-additive genetic models (OR = 1.60, CI 1.12–2.29, *p* = 0.010). Among the participants with BMI > 24, rs7914287 can also significantly increase the T2D risk under allele (OR = 1.45, CI 1.08–1.96, *p* = 0.014), homozygote (OR = 2.86, CI 1.32–6.24, *p* = 0.008), recessive (OR = 2.58, CI 1.22–4.47, *p* = 0.014), and log-additive genetic models (OR = 1.48, CI 1.09–2.01, *p* = 0.013).

**Table 6:** The SNPs of *CTNNA3* associated with susceptibility of type 2 diabetes in the subgroup tests (BMI)

SNP ID	Model	Genotype	BMI			
			OR (95% CI)	*p*	OR (95% CI)	*p*
			≤ 24 (N = 326)		> 24 (N = 426)	
rs10822745	Allele	C/T	1.12 (0.81–1.54)	0.499	0.89 (0.68–1.17)	0.415
	Homozygote	CC/TT	1.43 (0.69–2.95)	0.332	0.77 (0.44–1.37)	0.375
	Heterozygote	CT	0.95 (0.58–1.56)	0.839	1.06 (0.68–1.67)	0.793
	Dominant	CC-CT/TT	1.04 (0.64–1.67)	0.881	0.97 (0.63–1.50)	0.905
	Recessive	CC/CT-TT	1.48 (0.77–2.85)	0.244	0.74 (0.45–1.22)	0.237
	Log-additive	–	1.13 (0.81–1.59)	0.465	0.90 (0.68–1.19)	0.448
rs7920624	Allele	A/T	1.05 (0.76–1.44)	0.763	1.12 (0.85–1.47)	0.430
	Homozygote	AA/TT	1.13 (0.56–2.26)	0.733	1.25 (0.72–2.17)	0.422
	Heterozygote	AT	1.16 (0.66–2.04)	0.610	1.28 (0.81–2.02)	0.294
	Dominant	AA-AT/TT	1.15 (0.67–1.99)	0.613	1.27 (0.83–1.95)	0.276
	Recessive	AA/AT-TT	1.01 (0.58–1.77)	0.963	1.07 (0.67–1.72)	0.765
	Log-additive	–	1.06 (0.75–1.51)	0.727	1.13 (0.86–1.49)	0.385
rs2441727	Allele	A/G	0.72 (0.48–1.08)	0.115	1.00 (0.70–1.41)	0.993
	Homozygote	AA/GG	1.10 (0.19–6.24)	0.913	1.32 (0.32–5.51)	0.705
	Heterozygote	AG/GG	0.64 (0.39–1.03)	0.068	0.96 (0.63–1.45)	0.829
	Dominant	AA-AG/GG	0.66 (0.41–1.06)	0.082	0.97 (0.65–1.46)	0.896
	Recessive	AA/AG-GG	1.30 (0.23–7.31)	0.765	1.34 (0.32–5.56)	0.689
	Log-additive	–	1.16 (0.77–1.74)	0.484	1.00 (0.69–1.45)	0.991
rs7914287	Allele	T/C	1.57 (1.11–2.24)	0.072	1.45 (1.08–1.96)	0.014*
	Homozygote	TT/CC	3.66 (1.43–5.36)	0.120	2.86 (1.32–6.24)	0.008*
	Heterozygote	TC/CC	1.27 (0.79–2.06)	0.325	1.24 (0.82–1.88)	0.302
	Dominant	TT-TC/CC	1.52 (0.96–2.42)	0.073	1.42 (0.96–2.11)	0.080
	Recessive	TT/TC-CC	2.25 (1.31–4.07)	0.093	2.58 (1.22–4.47)	0.014*
	Log-additive	–	1.60 (1.12–2.29)	0.110	1.48 (1.09–2.01)	0.013*

OR, odds ratio; CI, confidence interval

*p* < 0.05, bold text and '*' indicate statistical significance

“–” indicates Log-additive model

*No retinal degeneration and course of T2D* The results showed (Table 7) that rs7914287 was associated with T2D patients who have no retinal egeneration under multiple genetic models (allele: *p* = 0.022, homozygote: *p* = 0.004, recessive: *p* = 0.003, log-additive: *p* = 0.021). We also found that the candidate SNPs associated with the course of T2D were rs10822745 (allele: *p* = 0.022, homozygote: *p* = 0.017, recessive: *p* = 0.027, log-additive: *p* = 0.023), rs7920624 (allele: *p* = 0.030), and rs2441727 (allele: *p* = 0.030; heterozygote: *p* = 0.001; dominant: *p* = 0.001; log-additive: *p* = 0.003).

**Table 7:** The SNPs of *CTNNA3* associated with susceptibility of type 2 diabetes in the subgroup tests (no retinal degeneration and course of type 2 diabetes)

SNP ID	Model	Genotype	No retinal degeneration (No retinal degeneration in cases vs. ≤ control) (N = 741)		Course of type 2 diabetes (> 10 vs. ≤ 10 years) (N = 500)	
			OR (95% CI)	*p*	OR (95% CI)	*p*
rs10822745	Allele	C/T	1.07 (0.86–1.33)	0.552	0.74 (0.57–0.96)	0.022*
	Homozygote	CC/TT	1.07 (0.66–1.72)	0.795	0.49 (0.28–0.88)	0.017*
	Heterozygote	CT	1.30 (0.92–1.85)	0.139	0.83 (0.55–1.25)	0.368
	Dominant	CC-CT/TT	1.24 (0.89–1.74)	0.204	0.73 (0.49–1.09)	0.120
	Recessive	CC/CT-TT	0.90 (0.59–1.38)	0.631	0.55 (0.33–0.94)	0.027*
	Log-additive	–	1.07 (0.86–1.35)	0.540	0.73 (0.55–0.96)	0.023*
rs7920624	Allele	A/T	0.95 (0.76–1.18)	0.647	1.33 (1.03–1.72)	0.030*
	Homozygote	AA/TT	0.88 (0.55–1.41)	0.602	1.73 (1.00–3.01)	0.052
	Heterozygote	AT	1.29 (0.88–1.90)	0.187	1.50 (0.94–2.40)	0.091
	Dominant	AA-AT/TT	1.16 (0.80–1.67)	0.430	1.56 (1.00–2.45)	0.051
	Recessive	AA/AT-TT	0.74 (0.50–1.08)	0.118	1.30 (0.84–2.03)	0.238
	Log-additive	–	0.95 (0.76–1.19)	0.640	1.31 (1.00–1.73)	0.051
rs2441727	Allele	A/G	0.81 (0.61–1.08)	0.152	1.33 (1.03–1.72)	0.030*
	Homozygote	AA/GG	0.56 (0.20–1.54)	0.261	0.61 (0.22–1.68)	0.336
	Heterozygote	AG/GG	0.83 (0.59–1.16)	0.268	0.49 (0.32–0.76)	0.001*
	Dominant	AA-AG/GG	0.80 (0.58–1.11)	0.188	0.50 (0.33–0.76)	0.001*
	Recessive	AA/AG-GG	0.59 (0.22–1.64)	0.314	0.74 (0.27–2.04)	0.558
	Log-additive	–	0.80 (0.60–1.08)	0.140	0.59 (0.41–0.84)	0.003*
rs7914287	Allele	T/C	1.31 (1.04–1.66)	0.022*	1.10 (0.85–1.44)	0.466
	Homozygote	TT/CC	2.20 (1.29–3.75)	0.004*	1.23 (0.70–2.16)	0.479
	Heterozygote	TC/CC	1.07 (0.77–1.49)	0.682	0.89 (0.60–1.34)	0.587
	Dominant	TT-TC/CC	1.23 (0.90–1.68)	0.200	0.97 (0.66–1.41)	0.859
	Recessive	TT/TC-CC	2.13 (1.28–3.54)	0.003*	1.30 (0.76–2.21)	0.334
	Log-additive	–	1.32 (1.04–1.68)	0.021*	1.05 (0.80–1.37)	0.721

OR, odds ratio; CI, confidence interval

*p* < 0.05, bold text and '*' indicate statistical significance

“–” indicates Log-additive model

*Differences in clinical indicators under different genotypes* We also evaluated the impact of 4 candidate CTNNA3 SNPs on the level of clinical indicators under different genotypes. The result showed (Table 8) that high density lipoprotein levels had significant differences under different genotypes of CTNNA3 rs10822745 (*p* = 0.013). The level of aspartate aminotransferase (*p* = 0.037), alanine aminotransferase (*p* = 0.044) and gamma-glutamyltransferase (*p* = 0.029) also had significant differences under different genotypes of rs7914287. There was no significant difference between the remaining candidate SNPs and the level of clinical indicators (Additional file 1: Table S1).

**Table 8:** Clinical characteristics of patients (N = 501) based on the genotypes of selected SNPs

Characteristics	rs10822745				rs7914287			
	TT	TC	CC	*p*	TT	TC	CC	*p*
FBS	7.22 ± 2.93	7.46 ± 3.46	7.23 ± 4.01	0.770	7.34 ± 3.03	7.31 ± 3.44	7.39 ± 3.48	0.976
GHbA1c	8.11 ± 1.99	8.17 ± 2.16	7.71 ± 1.83	0.212	7.6 ± 1.79	8.18 ± 1.99	8.11 ± 2.2	0.135
TC	3.5 ± 1.48	3.57 ± 1.48	3.73 ± 4.43	0.763	3.32 ± 1.59	3.64 ± 3.03	3.59 ± 1.47	0.598
TG	2.89 ± 3.41	2.66 ± 2.68	2.57 ± 2.41	0.670	2.44 ± 3.55	2.83 ± 2.6	2.69 ± 2.97	0.651
HDL	1.03 ± 0.28	0.99 ± 0.24	1.26 ± 1.63	0.013*	1.08 ± 0.33	1.1 ± 1.04	0.99 ± 0.25	0.306
Urea	6.36 ± 2.39	6.76 ± 3.94	6.1 ± 2.19	0.206	6.27 ± 1.71	6.4 ± 2.44	6.72 ± 4.21	0.494
Cr	70.78 ± 55.46	73.38 ± 56.92	65.54 ± 29.61	0.493	68.02 ± 29.28	68.17 ± 26.33	75.44 ± 74.01	0.320
Cys-c	0.99 ± 0.47	1.02 ± 0.48	1 ± 0.34	0.709	0.98 ± 0.38	1.03 ± 0.34	1 ± 0.57	0.690
AST	21.57 ± 13.08	22.01 ± 16.46	21.49 ± 11.82	0.939	22.62 ± 12.84	23.41 ± 19.17	19.84 ± 8.59	0.037*
ALT	24.68 ± 24.87	24.62 ± 27.39	25.22 ± 23.21	0.982	23.08 ± 14.14	28.07 ± 34.91	21.97 ± 15.68	0.044*
GGT	30.88 ± 24.8	34.61 ± 39.41	32.67 ± 57.54	0.652	29.63 ± 19.1	38.54 ± 54.75	28.72 ± 20.7	0.029*
LPa	205.49 ± 210.61	214.26 ± 223.75	246.77 ± 241.11	0.392	199.82 ± 199.91	230.56 ± 219.36	209.44 ± 233.49	0.516

*p* <0.05, bold text and ‘*’ represent statistical significance

FBS, fasting blood glucose; GHbA1c, glycosylated hemoglobin A1c; TC, total cholesterol; TG, triacylglycerol; HDL, high density lipoprotein; Cr, creatinine; Cys-c, cystatin c; AST, aspartate aminotransferase; ALT, alanine aminotransferase; GGT, gamma-glutamyltransferase; LPa, lysophosphatidic acid

### FPRP analysis

The results of FPRP analysis showed that (Additional file 2: Table S2) the association between *CTNNA3* rs7914287 and T2D risk in drinking participants was not noteworthy at the prior probability level of 0.25 and FPRP threshold of 0.2. The FPRP of the remaining significant results were all less than 0.2, which means that these positive results were noteworthy.

### LD and haplotype analysis

**Fig. 1:**
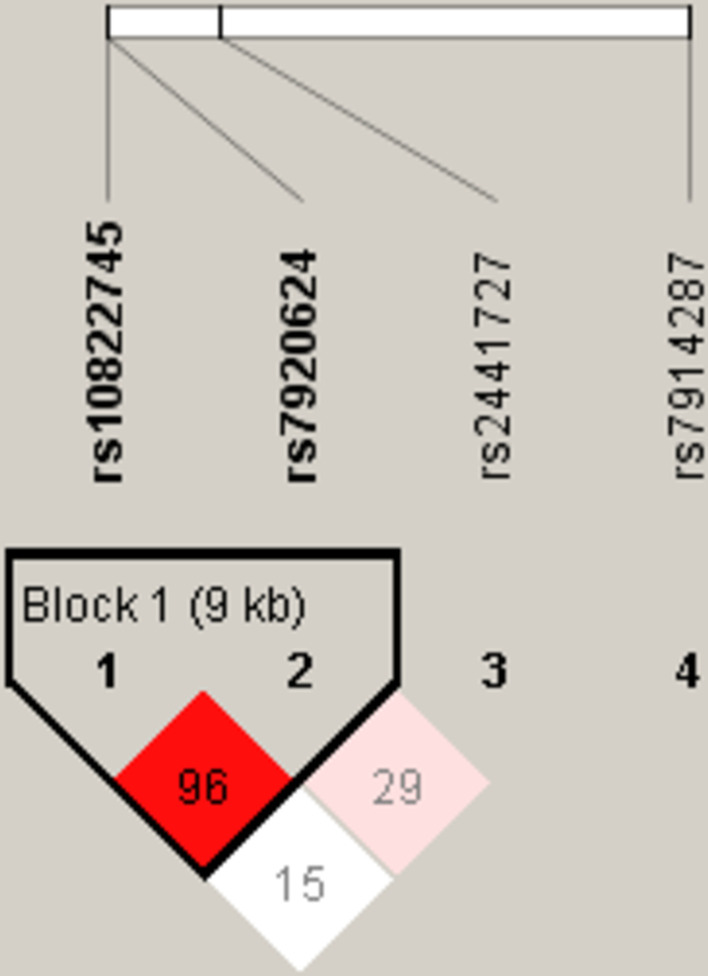
Linkage disequilibrium (LD) plots containing four polymorphisms from CTNNA3. The lighter the color, the lower the degree of linkage. The numbers inside the diamonds indicate the D′ for pairwise analyses

The results of linkage disequilibrium and haplotype analysis of *CTNNA3* polymorphism showed (Fig. 1): there is an LD block (D’ = 0.968, R^2^ = 0.665) composed of 2 SNPs (rs10822745 and rs7920624). However, logistic regression results showed that there was no statistically significant difference among the *CTNNA3* haplotype frequencies in the cases and controls (Additional file 3: Table S3).

### Analysis of MDR

**Fig. 2:**
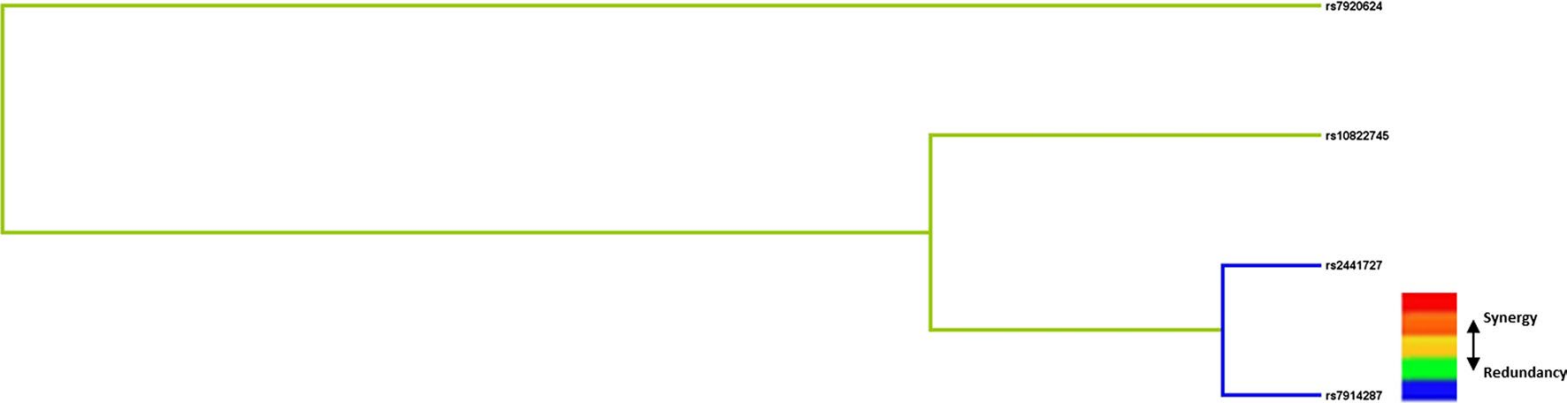
Dendrogram analysis of SNP-SNP interaction. The colors in the tree diagram represent synergy or redundancy

We used MDR to analyze and predict the interaction between SNP-SNP. Figure 2 was dendrogram analysis of SNP-SNP interaction. The color in the figure represents whether the effect of SNP-SNP on T2D risk is synergistic or redundant. The color in the figure represents whether the effect of SNP-SNP on T2D risk is synergistic or redundant. The blue line in the dendrogram indicates that candidate SNPs have a redundant role in regulating T2D risk (Fig. 2). The results (Table 9) showed that the four loci models (rs10822745, rs7920624, rs2441727, rs7914287) have the highest test accuracy. However, considering the small sample size, the rs7920624 and rs2441727 two-site model was regard as the overall best model, with a test accuracy of 0.541 and a good CVC (9/10).

**Table 9:** SNP–SNP interaction models analyzed by the MDR method

Model	Training Bal. Acc	Testing Bal. Acc	OR (95% CI)	*p* value	CVC
rs7914287	0.537	0.520	1.35 (1.05–1.73)	**0.0191**	9/10
rs7920624, rs2441727	0.557	0.541	1.58 (1.23–2.04)	**0.0003**	9/10
rs7920624, rs2441727, rs7914287	0.582	0.525	1.96 (1.52–2.54)	** < 0.0001**	9/10
rs10822745, rs7920624, rs2441727, rs7914287	0.607	0.543	2.41 (1.86–3.12)	** < 0.0001**	10/10

MDR, multifactor dimensionality reduction; Bal. Acc., balanced accuracy; CVC, cross-validation consistency; OR, odds ratio; 95% CI, 95% confidence interval

*p* values were calculated using χ^2^ tests;* p* < 0.05 and bold text indicate statistical significance

## Discussion

The incidence of Type 2 diabetes has increased significantly worldwide, and the number of T2D patients in many countries is increasing year by year [1]. More and more studies have confirmed that genetic factors play an indispensable role in the T2D risk [2]. More and more efforts are also devoted to improving the status quo of T2D, but there are still some clinical challenges to be overcome. For example, the existing clinical markers are not fully applicable to clinical diagnosis [25]. It was estimated that the genetic signals that have been discovered can only explain 2% of the T2D risk [16]. Therefore, it is still an arduous and long-term task to discover more genetic polymorphisms related to T2D risk.

There are relatively few studies on the association between *CTNNA3* genetic polymorphisms and diseases. In recent reports, *CTNNA3* single nucleotide polymorphisms in African populations can be used as new genetic signals for MetS, and MetS risk is closely associated with T2D risk [19, 20]. Our study is the first to explore the association between *CTNNA3* SNPs and T2D risk in Chinese Han population, and we found strong evidence of potential association between them. In general, among the 4 candidate SNPs, only *CTNNA3* rs7914287 was significantly associated with T2D risk in the Chinese Han population under allele, homozygous, recessive and log-additive models. It may be a risk factor for T2D. We found no evidence that the remaining three SNPs are associated with T2D risk among participants.

Type 2 diabetes is most common in the elderly, but due to lack of physical activity and healthy eating habits [26–28], there are more and more obese patients among children, adolescents and young people, which in turn leads to type 2 diabetes [29]. Therefore, we divide the participants according to the current status of T2D incidence and the potential risk factors of T2D for subgroup analysis, with a view to provide a valuable reference for T2D risk assessment in specific populations. And subgroup analysis for potential risk factors is an effective way to remove the influence of confounding factors. Alcohol [21], smoking [22], and aging [23] have been reported as risk factors for T2D. In this study, rs2441727 significantly reduced the T2D risk among participants who were > 60 years old, smoking, or drinking, and it also showed a trend to reduce the risk of T2D among participants who were ≤ 60 years old, no smoking/drinking. Our results indicate that *CTNNA3*-rs2441727 may be a protective factor for T2D in Chinese Han population, and this protective effect is not affected by the potential environmental risk factors of T2D. However, a large sample size and further verification tests are necessary to ensure that our results are more accurate.

Nevertheless, our study is the first to report the correlation between *CTNNA3*-rs2441727 and T2D risk.

Whether in the overall or subgroup analysis (age > 60 years old, smoking, drinking, male, BMI > 24, and no retinal degeneration), *CTNNA3*-rs7914287 can increase the T2D risk under multiple genetic models among participants. However, it is worth noting that in the overall analysis, under the allelic inheritance model, the allele ‘T’ of rs7914287 seemed to show a tendency to reduce the risk of T2D, the result was not significant. We speculated that potential risk factors such as age > 60 years old, males, smoking or drinking may promote the allele ‘T’ of rs7914287 to become a risk factor for T2D. In addition, we also found that the results of our study are similar to previous studies: Kautzky–Willer et al. have reported that T2D risk has gender differences. And this gender difference may be affected by environmental factors such as age and obesity rate [30]. Our study also found that the association between *CTNNA3*-rs7914287 and T2D risk had gender differences. Increased BMI is strongly associated with T2D risk [31]. Our study also found that rs7914287 was significantly associated with T2D risk among participants with BMI > 24. In addition to the above findings, we found that *CTNNA3*-rs7914287 was a risk factor for T2D patients with no retinal degeneration. However, numerous studies have reported that retinal degeneration is closely related to T2D [32, 33]. Combined with the results of this study, *CTNNA3*-rs7914287 can significantly increase the risk of T2D participants and may not be affected by retinal degeneration or not.

In summary, rs7914287 is a risk factor for T2D. And potential risk factors such as age > 60 years old, males, smoking or drinking et al. may have a synergistic effect with rs7914287 in increasing the risk of T2D.

And we were also pleasantly surprised to find that the levels of AST, ALT and GGT of T2D patients were significantly different under different genotypes of rs7914287. And there were studies have reported that the increase in AST, ALT and GGT levels may be related to the increased risk of T2D [32, 34, 35]. In our study, the T2D participants had significantly higher AST, ALT and GGT levels under the rs7914287 TC genotype. Therefore, we speculate that *CTNNA3*-rs7914287 may increase the T2D risk by affecting the levels of AST, ALT and GGT. But this is just a speculation, *CTNNA3*-rs7914287 mechanism in the pathogenesis of T2D risk remains unclear, further research is needed. Nevertheless, our study suggest that *CTNNA3* genetic polymorphism may be a new genetic signal of T2D risk in Chinese Han population, providing new ideas and valuable references for clinical early prevention and individualized treatment of T2D in Chinese Han population.

It is worth noting that our study still has certain limitations. If the sample size is further expanded for research verification, it will be more helpful to confirm the results of our study.

## Conclusion

In summary, we found that *CTNNA3* genetic polymorphisms can be used as a new genetic signal of T2D risk in Chinese Han population. Especially, *CTNNA3*-rs7914287 showed an outstanding and significant association with T2D risk in both overall analysis and subgroup analysis. Our study has provided valuable data supplements for the T2D susceptibility loci in Chinese Han population.

## Supplementary Information

**Additional file 1. Supplemental table 1** Clinical characteristics of patients based on the genotypes of selected SNPs.

**Additional file 2. Supplementary table 2** The FPRP and statistical power values of the positive results in this study.

**Additional file 3. Supplemental table 3** Haplotype frequencies and the association with the risk of T2D.

## Data Availability

The datasets generated and/or analysed during the current study are available in the [Zenodo] repository, https://doi.org/10.5281/zenodo.5251076.
