# Supplementary material for: CTNNA3 genetic polymorphism may be a new genetic signal of type 2 diabetes in the Chinese Han population: a case control study
Source: BMC Med Genomics. 2021 Oct 30;14:257. doi: 10.1186/s12920-021-01105-8 (PMC8556947; doi:10.1186/s12920-021-01105-8)
Supplement: Supplementary file 1 — Additional file 1. Supplemental table 1 Clinical characteristics of patients based on the genotypes of selected SNPs. [file 12920_2021_1105_MOESM1_ESM.docx]

**Additional file 1: Table S1** Clinical characteristics of patients based on the genotypes of selected SNPs.

| **Characteristics** | **rs7920624** | | | |  | **rs2441727** | | | |
| --- | --- | --- | --- | --- | --- | --- | --- | --- | --- |
|  | **AA** | **AT** | **TT** | ***p*** |  | **AA** | **AG** | **GG** | ***p*** |
| FBS | 7.07 ± 2.65 | 7.48 ± 3.51 | 7.23 ± 3.73 | 0.562 |  | 6.54 ± 3.62 | 7.63 ± 3.73 | 7.26 ± 3.23 | 0.358 |
| GHbA1c | 7.92 ± 1.78 | 8.25 ± 2.21 | 7.78 ± 1.83 | 0.094 |  | 7.72 ± 2.22 | 8.08 ± 1.95 | 8.08 ± 2.1 | 0.788 |
| TC | 3.42 ± 1.46 | 3.59 ± 1.49 | 3.68 ± 3.82 | 0.672 |  | 2.66 ± 1.32 | 3.69 ± 1.49 | 3.57 ± 2.6 | 0.215 |
| TG | 2.63 ± 2.73 | 2.85 ± 3.21 | 2.55 ± 2.29 | 0.625 |  | 2.93 ± 2.6 | 2.48 ± 2.21 | 2.81 ± 3.16 | 0.507 |
| HDL | 1 ± 0.26 | 1.01 ± 0.25 | 1.18 ± 1.38 | 0.089 |  | 1.03 ± 0.21 | 1.09 ± 0.69 | 1.03 ± 0.75 | 0.715 |
| Urea | 6.59 ± 2.6 | 6.75 ± 3.98 | 5.96 ± 1.68 | 0.090 |  | 6.67 ± 3.37 | 6.27 ± 2.59 | 6.62 ± 3.51 | 0.553 |
| Cr | 75.65 ± 64.68 | 72.58 ± 57.37 | 64.64 ± 22.19 | 0.251 |  | 76.82 ± 40.83 | 66.36 ± 27.08 | 73.02 ± 61.02 | 0.409 |
| Cys-c | 1.03 ± 0.54 | 1.02 ± 0.49 | 0.96 ± 0.29 | 0.485 |  | 1.19 ± 0.42 | 0.96 ± 0.34 | 1.02 ± 0.5 | 0.089 |
| AST | 21.8 ± 14.54 | 21.71 ± 15.87 | 21.9 ± 12.38 | 0.993 |  | 22.06 ± 11.86 | 20.95 ± 9.95 | 22.13 ± 16.48 | 0.717 |
| ALT | 24.41 ± 27.79 | 24.67 ± 26.81 | 25.08 ± 22.37 | 0.980 |  | 19.35 ± 15.52 | 24.55 ± 17.24 | 25.11 ± 29.27 | 0.667 |
| GGT | 32 ± 26.23 | 33.74 ± 38.54 | 33.13 ± 50.81 | 0.930 |  | 34.47 ± 25.33 | 31.21 ± 24.73 | 33.88 ± 45.13 | 0.787 |
| LPa | 229.54 ± 235.74 | 209.83 ± 218.11 | 226.33 ± 223.61 | 0.688 |  | 220.47 ± 154.78 | 222.17 ± 235.42 | 214.72 ± 220.61 | 0.947 |

FBS: fasting blood glucose; GHbA1c: glycosylated hemoglobin A1c; TC: total cholesterol; TG: triacylglycerol; HDL: high density lipoprotein; Cr: creatinine; Cys-c: cystatin c; AST: aspartate aminotransferase; ALT: alanine aminotransferase; GGT: gamma-glutamyltransferase; LPa: lysophosphatidic acid.
