# Supplementary material for: CTNNA3 genetic polymorphism may be a new genetic signal of type 2 diabetes in the Chinese Han population: a case control study
Source: BMC Med Genomics. 2021 Oct 30;14:257. doi: 10.1186/s12920-021-01105-8 (PMC8556947; doi:10.1186/s12920-021-01105-8)
Supplement: Supplementary file 2 — Additional file 2. Supplementary table 2 The FPRP and statistical power values of the positive results in this study. [file 12920_2021_1105_MOESM2_ESM.docx]

**Additional file 2: Table S2** The FPRP and statistical power values of the positive results in this study.

| SNP ID | Model | Genotype | OR (95%CI) | *p* | Statistical Power ^a^ (%) | Prior probability | | |
| --- | --- | --- | --- | --- | --- | --- | --- | --- |
|  |  |  |  |  |  | 0.25 | 0.1 | 0.01 |
| **Overall analysis** | | | | | | | | |
| rs7914287 | Allele | T/C | 1.33(1.10-1.61) | 0.003 | 100 | 0.010 ^b^ | 0.030 ^b^ | 0.254 |
|  | Genotype | TT/CC | 2.21(1.41-3.47) | 0.001 | 33.2 | 0.005 ^b^ | 0.015 ^b^ | 0.145 ^b^ |
|  | Recessive | TT/TC-CC | 2.09(1.36-3.22) | 0.001 | 42.1 | 0.006 ^b^ | 0.017 ^b^ | 0.163 ^b^ |
|  | Log-additive | - | 1.34(1.10 – 1.62) | 0.003 | 100.0 | 0.007 ^b^ | 0.022 ^b^ | 0.199 ^b^ |
| **Subgroup analysis: age (age > 60)** | | | | | | | | |
| rs2441727 | Genotype | AG/GG | 0.58(0.39-0.87) | 0.008 | 76.3 | 0.032 ^b^ | 0.091 ^b^ | 0.523 |
|  | Dominant | AA-AG/GG | 0.64(0.44-0.93) | 0.021 | 90.2 | 0.060 ^b^ | 0.161 | 0.679 |
| rs7914287 | Allele | T/C | 1.37(1.06-1.79) | 0.018 | 99.7 | 0.059 ^b^ | 0.160 ^b^ | 0.676 |
|  | Genotype | TT/CC | 2.22(1.18-4.17) | 0.013 | 37.3 | 0.096 ^b^ | 0.241 | 0.777 |
|  | Dominant | TT-TC/CC | 1.56(1.08-2.25) | 0.019 | 90.8 | 0.054 ^b^ | 0.147 ^b^ | 0.654 |
|  | Recessive | TT/TC-CC | 1.85(1.02-3.35) | 0.044 | 60.2 | 0.174 ^b^ | 0.388 | 0.874 |
|  | Log-additive | - | 1.47(1.11-1.94) | 0.007 | 98.5 | 0.019 ^b^ | 0.056 ^b^ | 0.395 |
| **Subgroup analysis: gender (male)** | | | | | | | | |
| rs7914287 | Allele | T/C | 2.33(1.07-3.67) | 0.012 | 25.5 | 0.003 ^b^ | 0.009 ^b^ | 0.093 ^b^ |
|  | Genotype | TT/CC | 2.20(1.29-3.77) | 0.004 | 36.4 | 0.033 ^b^ | 0.092 ^b^ | 0.528 |
|  | Recessive | TT/TC-CC | 2.08(1.24-3.49) | 0.005 | 44.1 | 0.036 ^b^ | 0.102 ^b^ | 0.554 |
|  | Log-additive | - | 2.33(1.06-3.67) | 0.013 | 25.5 | 0.003 ^b^ | 0.009 ^b^ | 0.093 ^b^ |
| **Subgroup analysis: smoking (Yes)** | | | | | | | | |
| rs2441727 | Genotype | AG/GG | 0.61(0.41-0.92) | 0.018 | 82.9 | 0.062 ^b^ | 0.166 ^b^ | 0.687 |
|  | Dominant | AA-AG/GG | 0.65(0.44-0.97) | 0.034 | 90.1 | 0.104 ^b^ | 0.259 | 0.793 |
| rs7914287 | Allele | T/C | 2.36(1.01-4.82) | 0.023 | 32.5 | 0.146 ^b^ | 0.338 | 0.849 |
|  | Genotype | TT/CC | 2.50(1.18-5.32) | 0.017 | 28.1 | 0.157 ^b^ | 0.358 | 0.860 |
|  | Recessive | TT/TC-CC | 2.44(1.18-5.05) | 0.016 | 29.6 | 0.141 ^b^ | 0.330 | 0.844 |
| **Subgroup analysis: drinking (Yes)** | | | | | | | | |
| rs2441727 | Genotype | AG/GG | 0.63(0.42-0.94) | 0.025 | 87.1 | 0.075 ^b^ | 0.196 ^b^ | 0.729 |
| rs7914287 | Allele | T/C | 1.41(1.04-2.91) | 0.025 | 82.8 | 0.561 | 0.793 | 0.977 |
|  | Genotype | TT/CC | 2.11(1.34-5.20) | 0.008 | 45.4 | 0.409 | 0.675 | 0.958 |
|  | Recessive | TT/TC-CC | 2.00(1.33-4.77) | 0.008 | 50.0 | 0.415 | 0.680 | 0.959 |
| **Subgroup analysis: BMI (BMI > 24)** | | | | | | | | |
| rs7914287 | Allele | T/C | 1.45(1.08-1.96) | 0.014 | 98.2 | 0.046 ^b^ | 0.126 ^b^ | 0.612 |
|  | Genotype | TT/CC | 2.86(1.32-6.24) | 0.008 | 18.4 | 0.119 ^b^ | 0.288 | 0.817 |
|  | Recessive | TT/TC-CC | 2.58(1.22-4.47) | 0.014 | 18.2 | 0.012 ^b^ | 0.035 ^b^ | 0.283 |
|  | Log-additive | - | 1.48(1.09-2.01) | 0.013 | 97.3 | 0.036 ^b^ | 0.100 ^b^ | 0.551 |
| **Subgroup analysis:** **no** **retinal degeneration** | | | | | | | | |
| rs7914287 | Allele | T/C | 1.31(1.04-1.66) | 0.022 | 100.0 | 0.071 ^b^ | 0.186 ^b^ | 0.716 |
|  | Genotype | TT/CC | 2.20(1.29-3.75) | 0.004 | 36.3 | 0.030 ^b^ | 0.085 ^b^ | 0.506 |
|  | Recessive | TT/TC-CC | 2.13(1.28-3.54) | 0.003 | 40.4 | 0.026 ^b^ | 0.073 ^b^ | 0.464 |
|  | Log-additive | - | 1.32(1.04-1.68) | 0.021 | 100.0 | 0.067 ^b^ | 0.178 ^b^ | 0.704 |
| **Subgroup analysis:** **course of type 2 diabetes** | | | | | | | | |
| rs10822745 | Allele | C/T | 0.74(0.57-0.96) | 0.022 | 99.8 | 0.066 ^b^ | 0.174 ^b^ | 0.699 |
|  | Genotype | CC/TT | 0.49(0.28-0.88) | 0.017 | 47.3 | 0.097 ^b^ | 0.244 | 0.780 |
|  | Recessive | CC/CT-TT | 0.55(0.33-0.94) | 0.027 | 63.6 | 0.120 ^b^ | 0.289 | 0.818 |
|  | Log-additive | - | 0.73(0.55-0.96) | 0.023 | 99.7 | 0.068 ^b^ | 0.180 ^b^ | 0.707 |
| rs7920624 | Allele | A/T | 1.33(1.03-1.72) | 0.030 | 99.9 | 0.082 ^b^ | 0.211 | 0.747 |
| rs2441727 | Allele | A/G | 1.33(1.03-1.72) | 0.030 | 99.9 | 0.082 ^b^ | 0.211 | 0.747 |
|  | Genotype | AG/GG | 0.49(0.32-0.76) | 0.001 | 46.4 | 0.009 ^b^ | 0.027 ^b^ | 0.236 |
|  | Dominant | AA-AG/GG | 0.50(0.33-0.76) | 0.001 | 50.0 | 0.007 ^b^ | 0.021 ^b^ | 0.189 |
|  | Log-additive | - | 0.59(0.41-0.84) | 0.003 | 82.1 | 0.012 ^b^ | 0.036 ^b^ | 0.292 |

FPRP: false-positive report probability.

^a^ Statistical power ^a^ was calculated using the number of the OR and p values in this table.

^b^ The level of false-positive report probability threshold was set at 0.2, and noteworthy findings are presented.
