# Supplementary material for: CTNNA3 genetic polymorphism may be a new genetic signal of type 2 diabetes in the Chinese Han population: a case control study
Source: BMC Med Genomics. 2021 Oct 30;14:257. doi: 10.1186/s12920-021-01105-8 (PMC8556947; doi:10.1186/s12920-021-01105-8)
Supplement: Supplementary file 3 — Additional file 3. Supplemental table 3 Haplotype frequencies and the association with the risk of T2D. [file 12920_2021_1105_MOESM3_ESM.docx]

**Additional file 3: Table S3** Haplotype frequencies and the association with the risk of T2D.

| **Chromosome** | **Gene** | **SNP** | **haplotype** | **Frequency** | | **Crude analysis** | | **Adjusted by age and gender** | |
| --- | --- | --- | --- | --- | --- | --- | --- | --- | --- |
|  |  |  |  | **Cases** | **Controls** | **OR (95%CI)** | ***p*** | **OR (95%CI)** | ***p*** |
| Chr2 | CTNNA3 | rs10822745\|rs7920624 | TA | 0.485 | 0.491 | 1.00 | - | 1.00 | - |
|  |  | rs10822745\|rs7920624 | CT | 0.427 | 0.410 | 1.05 (0.87-1.27) | 0.600 | 1.05 (0.87 - 1.27) | 0.610 |
|  |  | rs10822745\|rs7920624 | TT | 0.086 | 0.092 | 0.95 (0.69 - 1.31) | 0.740 | 0.94 (0.68 - 1.30) | 0.730 |

SNP, single nucleotide polymorphism;

OR, Odds ratio;

CI, Confidence interval;

*p* < 0.05 indicates statistical significance.
